# Magnetic-activated cell sorting identifies a unique lung microbiome community

**DOI:** 10.1186/s40168-022-01434-5

**Published:** 2023-05-25

**Authors:** Daniel G. Dunlap, Libing Yang, Shulin Qin, Kelvin Li, Adam Fitch, Laurence Huang, Bryan J. McVerry, Timothy W. Hand, Barbara A. Methé, Alison Morris

**Affiliations:** 1 Division of Pulmonary, Allergy and Critical Care Medicine, Department of Medicine, University of Pittsburgh School of Medicine and University of Pittsburgh Medical Center, NW628, 3459 Fifth Avenue, Pittsburgh, PA 15213 USA; 2 Center for Medicine and the Microbiome, University of Pittsburgh, Pittsburgh, USA; 3 Department of Medicine, University of California, San Francisco, CA USA; 4 Children’s Hospital of Pittsburgh, Pittsburgh, PA USA; 5 Department of Immunology, University of Pittsburgh School of Medicine, Pittsburgh, PA USA

**Keywords:** Microbiome, Immunoglobulin, Host response

## Abstract

**Background:**

The advent of culture-independent, next-generation DNA sequencing has led to the discovery of distinct lung bacterial communities. Studies of lung microbiome taxonomy often reveal only subtle differences between health and disease, but host recognition and response may distinguish the members of similar bacterial communities in different populations. Magnetic-activated cell sorting has been applied to the gut microbiome to identify the numbers and types of bacteria eliciting a humoral response. We adapted this technique to examine the populations of immunoglobulin-bound bacteria in the lung.

**Methods:**

Sixty-four individuals underwent bronchoalveolar lavage (BAL). We separated immunoglobulin G-bound bacteria using magnetic-activated cell sorting and sequenced the 16S rRNA gene on the Illumina MiSeq platform. We compared microbial sequencing data in IgG-bound bacterial communities compared to raw BAL then examined the differences in individuals with and without HIV as a representative disease state.

**Results:**

Immunoglobulin G-bound bacteria were identified in all individuals. The community structure differed when compared to raw BAL, and there was a greater abundance of *Pseudomonas* and fewer oral bacteria in IgG-bound BAL. Examination of IgG-bound communities in individuals with HIV demonstrated the differences in Ig-bound bacteria by HIV status that were not seen in a comparison of raw BAL, and greater numbers of immunoglobulin-bound bacteria were associated with higher pulmonary cytokine levels.

**Conclusions:**

We report a novel application of magnetic-activated cell sorting to identify immunoglobulin G-bound bacteria in the lung. This technique identified distinct bacterial communities which differed in composition from raw bronchoalveolar lavage, revealing the differences not detected by traditional analyses. Cytokine response was also associated with differential immunoglobulin binding of lung bacteria, suggesting the functional importance of these communities.

Video Abstract

**Supplementary Information:**

The online version contains supplementary material available at 10.1186/s40168-022-01434-5.

## Background

Studying the lung microbiome is challenging given its low biomass, the propensity to detect contaminating bacteria using next-generation sequencing techniques, and the inability to determine the functional impact of pulmonary microbes [1, 2]. As a result, studies that compare the lung microbiome in different diseases such as chronic obstructive pulmonary disease (COPD) or human immunodeficiency virus (HIV) infection often have conflicting conclusions or find few major differences between bacterial communities [3].

Magnetic-activated cell sorting (MACS) can be used to separate and identify immunoglobulin-bound bacteria. Bacteria are stained with fluorophores specific for bacterial DNA and the Fc region of any immunoglobulin adhered to the bacterial cell wall. Magnetic labeling of stained bacteria allows immunoglobulin G (IgG)-bound and unbound bacteria to be separated and analyzed. This technique has been applied to studies of the gut microbiome, and Ig-bound bacteria have been found to modulate inflammatory bowel disease [4, 5]. This technique has not been applied to the lung, but could serve to distinguish bacteria provoking an immune response versus those that are merely “innocent bystanders.”

We adapted the MACS technique from gut studies and applied it to the lung using bronchoalveolar lavage (BAL) to determine if IgG-bound bacterial communities differ from unsorted (raw) bacteria. First, we performed the MACS assay on BAL obtained from healthy individuals, with no known underlying lung pathology or chronic illness. We performed quantitative polymerase chain reaction (qPCR) and next-generation sequencing on the 16S rRNA gene in IgG-bound and unsorted (raw) BAL samples. We also used people living with human immunodeficiency virus (HIV) as a representative cohort to evaluate the application of this technique to a disease state in which humoral recognition and response to the lung microbiome may be contributing to the pathogenesis of chronic lung disease. Isolating immunoglobulin-bound bacteria from the lung is a novel way of investigating the microbiome’s impact on pulmonary health and disease .

## Methods

### Study participants

We utilized the University of Pittsburgh HIV research study at the Pittsburgh, Los Angeles, and San Francisco sites which includes both HIV-uninfected controls as well as people living with HIV. Subjects had no acute respiratory symptoms at the time of enrollment and were specifically without fevers, upper respiratory infection, or lung infection. We excluded individuals who had received systemic corticosteroids within 6 months of bronchoscopy and individuals receiving antibiotic therapy within 3 months of participation. Individuals participated in a variety of studies of pulmonary disease in HIV [6], and we included those with sufficient volumes of BAL for evaluation. All participants provided written informed consent. The University of Pittsburgh, University of California, Los Angeles, and University of California, San Francisco, institutional review boards approved this study.

### Sample collection

We collected lung samples via bronchoscopy as previously described [6]. Participants were instructed to avoid any food or beverages beginning at midnight before bronchoscopy and abstain from smoking at least 12 h prior to the procedure. All subjects gargled an antiseptic just prior to instrumentation to limit bacterial contamination from the oropharynx. Before bronchoscopy, 10 to 50 mL 0.9% normal saline was flushed through the bronchoscope and used as a negative control. Under moderate sedation, the bronchoscope was passed through the vocal cords and quickly placed into a wedge position in the right middle lobe. We collected BAL using sterile 0.9% normal saline to a maximal instillation volume of 200 mL. BAL specimens were immediately fractioned into 1- and 5-mL aliquots and stored at − 80 °C.

### Magnetic-activated cell sorting

One milliliter BAL and 1 mL buffer (1% BSA in PBS) were filtered into 2-mL microcentrifuge tubes, and bacteria were isolated via centrifugation (5000*g* × 5 min at 4 °C). We re-suspended bacterial pellets in the buffer and removed a small amount of solution to be used in compensation controls for flow cytometry performed at the conclusion of each sorting assay (described below). Following an additional centrifugation, the remaining bacterial pellets were suspended in a solution containing the bacterial DNA stain, SytoBC (Thermo Fisher Scientific, Waltham, MA), and the immunoglobulin stain, IgG-PE (eBioscience, Thermo Fisher Scientific, Waltham, MA). We incubated the stained bacteria with anti-PE microbeads (Miltenyi Biotec, USA) and then used centrifugation to condense magnetically labeled, stained bacteria prior to magnetic-activated cell sorting (MACS) before re-suspending in the buffer.

After washing MACS columns (Miltenyi Biotec) with buffer, we instilled individual bacterial samples into corresponding MACS columns that were embedded within a supermagnet (OctoMACS Separator, Miltenyi Biotec). Fifteen milliliters of conicals (IgG-unbound) was stationed beneath each column. Once the entire suspended bacterial sample passed through, each column was removed from the supermagnet, directed into its respective, designated conical (IgG-bound), and flushed with 1 mL buffer. The buffer was vigorously plunged through the column, per the manufacturer’s instructions, removing the magnetically labeled material that had been held in suspension during the assay. The columns were then placed atop a vacuum manifold (Promega, Madison WI) where they were washed with 70% ethanol followed by the buffer. The columns were replaced to their prior locations within OctoMACS Separator, and the contents within the IgG-unbound conicals were instilled into their respective columns, directly above the now empty IgG-unbound conicals repositioned below. An additional washing step followed, with another 1-mL buffer plunged through the column into the same IgG-bound conical, bringing the total volume within each conical (IgG-bound and IgG-unbound) to 2 mL, 450 μL of which was removed from each conical and used for flow cytometry analysis.

### Flow cytometry

We performed flow cytometry immediately following each MACS assay. Four hundred fifty microliters from IgG-bound and IgG-unbound aliquots was stored at 4 °C prior to flow cytometry analysis. After performing stain compensation on the STI Fortessa (STI Electronics, Madison, AL), all samples were run at high-throughput speed for 4 min. Plotting side scatter (SCC-A) against fluorescein isothiocyanate (FITC, emission 488nm) was used to gate the appropriate population of interest. A second plot of FITC vs phycoerythrin (PE, emission 578 nm) was performed. Gated reads with both a positive FITC and PE signal (> 10^3^) were quantified, representing the relative quantity of IgG-bound bacteria in each sample to confirm appropriate IgG sorting during MACS (Additional file 1: Fig. S1). Negative column controls, which contained only processed, stained buffer, were performed during sample runs as a quality control to confirm adequate sorting and used for subsequent 16S analysis.

### Quantitative PCR

Quantitative PCR amplification was performed in a total volume of 20-μL amplification reaction consisting of 2 μL of 10× PCR buffer, 3.5 mmol/L MgCl_2_, 0.2 mmol/L deoxynucleoside triphosphate, 0.5 μmol/L forward and reverse primers, 0.225 μmol/L probe, 0.75 U of Platinum Taq polymerase (Invitrogen), and 2 μL of each DNA. The forward and reverse primers were used to amplify DNA templates encoding 16S rRNA and both primer, and probe sequences were identical to those previously described [7]. A standard curve was created from serial dilutions of plasmid DNA containing known copy numbers of the template. The assays were performed on the LightCycler System (Roche) using the following PCR conditions: 95 °C for 5 min, followed by 50 cycles at 95 °C for 15 s and at 60 °C for 1 min.

### Luminex panels and cytokine analysis

We used a commercially available Luminex assay (Bio-Plex Pro™ Human Cytokine 17-plex Assay, Bio-Rad Laboratories, Hercules CA) to quantify cytokines present within BAL, as per the manufacturer’s instructions. The cytokines used for analysis included interleukin (IL)-1β, IL-2, IL-6, IL-8, macrophage inflammatory protein (MIP-1B, also known as CCL4), interferon (IFN)-γ, and tumor necrosis factor (TNF)-α. No dilutions were performed.

### Sample processing and sequencing

We extracted bacterial DNA from IgG-bound, IgG-unbound, and unsorted (raw) BAL samples using DNeasy PowerSoil Kits (Qiagen, Germantown MD) as per the manufacturer’s instructions then amplified the V4 subunit of the bacterial 16S gene using polymerase chain reaction (PCR). We performed sequencing on the Illumina MiSeq platform [8] and de-multiplexed reads onboard the machine using standard Illumina software. We then performed post-sequencing quality control (including sequence filtering and trimming raw 16S rRNA gene sequence) using an in-house pipeline developed by the University of Pittsburgh Center for Medicine and the Microbiome, utilizing available software including fastx toolkit, cutadapt, and dust [9–11]. We generated operational taxonomic units (OTUs) at a 97% identity threshold and RDP-classified sequences using an in-house mothur pipeline [12, 13]. Contamination controls were performed during bronchoscopy, MACS, DNA extraction, and PCR. Distribution-based contaminant filtering was applied to column controls to remove background signal from MACS columns and beads (described below).

### Distribution-based contaminant filtering

We applied computational contaminant filtering to subtract the pattern of taxa found in the controls from the experimental samples. We estimated the proportion of each taxa deemed contaminant and then removed them from the experimental measurements. The proportion to remove follows this mixture model:

$${(1-\mathrm p)}^\ast\mathrm{actExp}+\mathrm p^\ast\mathrm{actCont}=\mathrm{obsExp}$$

Each observed experimental (obsExp) sample has its own proportion, *p*, of actual contaminant (actCont) and the remaining proportion (1 − *p*) of the actual experimental (actExp) sample. Since we cannot measure the actual contaminant, we use the observed controls (obsCont) as an approximation. Each sample is a multivariate measurement of taxa abundance and should be considered compositional. Thus, the mixture model assumes that the relative proportion of each taxa, introduced by the contaminant is fixed. By fitting this objective function, the parameter *p* can be estimated for each sample by minimizing this objective function.

$$\mathrm{Sum}{(\mathrm p\ast\mathrm{obsCont}\lbrack\mathrm i\rbrack-(1-\mathrm p)\ast\mathrm{obsExp}\lbrack\mathrm i\rbrack)^2}$$

In this equation, obsCont[*i*] and obsExp[*i*] represent the relative abundance of the *i*th taxon among the observed control and observed experimental sample, respectively. This sum of squared differences objective function is minimized when the scaled shape of the observed control’s distribution of taxa overlaps the shape of the observed experimental sample’s distribution. Since there is an expectation that the observed experimental (obsExp) sample will contain taxa not found in the observed control (obsCont), these taxa are excluded from the objective function. This ensures that there is no mismatch penalty for the observed experimental sample containing taxa not found in the observed control. The same proportion *p* is applied to each taxa for a given sample. If an observed experimental sample is purely contaminant, then the objective function will minimize to 0, because the distribution of taxa in the observed experimental sample will look identical to the observed control sample.

After the contaminant proportion *p* is estimated, the proportion of the observed control is subtracted from the observed experimental. If the subtraction yields a negative value for a particular taxa, then its new abundance is set to 0. The resultant distribution of taxa is then normalized so that the sum across the relative abundance of all taxa equals 1. The calculated proportion is also used to scale down the read depth, so that the actual number of filtered reads per sample can be estimated.

In this experiment, since a small batch of controls was measured for a large set of experimental samples, the average across available controls was used as the observed control since the variation across them was low and significantly different than the experimental samples. If a larger number of controls (> 20) or a specific control was collected for each experimental sample, then the closest or a specifically matching control to the experimental could be used as the observed control.

### Quantifying immunoglobulin levels

Both serum and BAL immunoglobulin G levels were quantified. Serum samples were sent to the University of Pittsburgh Medical Center Clinical Laboratory (3460 Fifth Ave., Pittsburgh, PA), which quantified levels using commercially available ELISA assays. To quantify BAL IgG levels, we used a commercially available ELISA assay (Human IgG ELISA Kit, Abcam, Cambridge, MA) using the manufacturer’s instructions, diluting BAL at a 1:500 ratio.

### Statistical analysis

We applied a non-metric multidimensional scaling plot (NMDS) using Manhattan distance to the visualize sample clustering and performed multivariate analysis of variance (PERMANOVA) to compare the microbiota by IgG status with the *R* vegan package. We performed paired testing of BAL samples between IgG-bound and unsorted samples using Wilcoxon paired *p*-values to detect the differences in the additive log-transformed (ALR) abundance of individual bacterial taxon. When controlling for sex, age, and smoking status, the difference in the ALR was modeled as a multivariate response in a multiple regression. Sixty-four IgG-bound, IgG-unbound, and raw samples were analyzed, though one raw sample had zero reads and was excluded from the subsequent 16S analysis. Sorted IgG-unbound aliquots largely resembled column contamination and were thus excluded from subsequent analysis (Additional file 1: Fig. S2). We then compared communities by HIV status. Cytokine levels were log-transformed and plotted against log-transformed IgG-bound bacteria and qPCR data, and a linear regression model was used to evaluate the association between the two variables.

## Results

### Comparison of IgG-bound lung microbiota to raw BAL microbiota

**Fig. 1:**
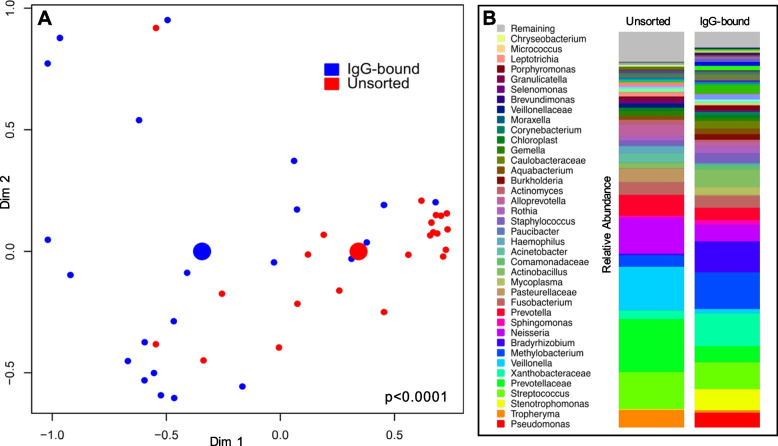
MACS identifies an IgG-bound lung microbiome that differs from raw BAL in healthy individuals. A non-metric multi-dimensional scaling plot (NMDS) (**A**) and stacked bar plots (**B**) detail the differences observed between the IgG-bound and raw lung microbiome in 22 individuals. **A** We applied an NMDS plot using Manhattan distance to visualize the sample clustering and performed a multivariate analysis of variance (PERMANOVA) to compare the beta diversity between IgG-bound and raw (unsorted) samples from 16S gene sequencing data. Points represent bacterial communities of individual samples, color-coded by IgG status (blue = IgG-bound, red = unsorted/raw). Larger colored centroids represent the mean group values. We found significant taxonomic differences between the groups (*p* < 0.0001, *R*^2^ = 0.09). **B** Stacked bar plots depict the relative abundance of bacteria in unsorted and IgG-bound BAL samples. In raw BAL samples, common oral microbes such as *Streptococcus*, *Prevotella*, and *Veillonella* were most abundant. The relative abundance of bacteria differed in the IgG-bound fraction

There were 22 control individuals with a mean age of 50.7 and a median pack-year smoking history of 6.2 years (Table 1). Bacterial relative abundance and overall community structure differed between IgG-bound and unsorted BAL samples (PERMANOVA for Manhattan distance, *p* < 0.0001, *R*^2^ = 0.09; Fig. 1A). While similar bacteria were seen in both unsorted and IgG-bound BAL samples, their relative abundance differed (Fig. 1B). *Streptococcus*, *Prevotella*, and *Veillonella* were more abundant in unsorted BAL samples relative to IgG-bound BAL (Additional file 1: Fig. S6). These oral microbes were also seen in IgG-bound samples, but *Pseudomonas* was detected in greater relative abundance when compared to raw BAL samples, though did not reach statistical significance (*p* = 0.12).

**Table 1:** Participant demographics among HIV-uninfected individuals (*n* = 22)

Age, mean (range)	50.7 (37–64)
Male, *n* (%)	15 (68.2)
Race, *n* (%)	
White	12 (54.5)
Black	7 (31.8)
Others	3 (13.6)
COPD, *n* (%)	8 (36.3)
Ever smoker, *n* (%)	12 (54.5)
Pack-years, median (range)	6.2 (0–54)

### Comparison of raw and IgG-bound BAL by HIV status

**Fig. 2:**
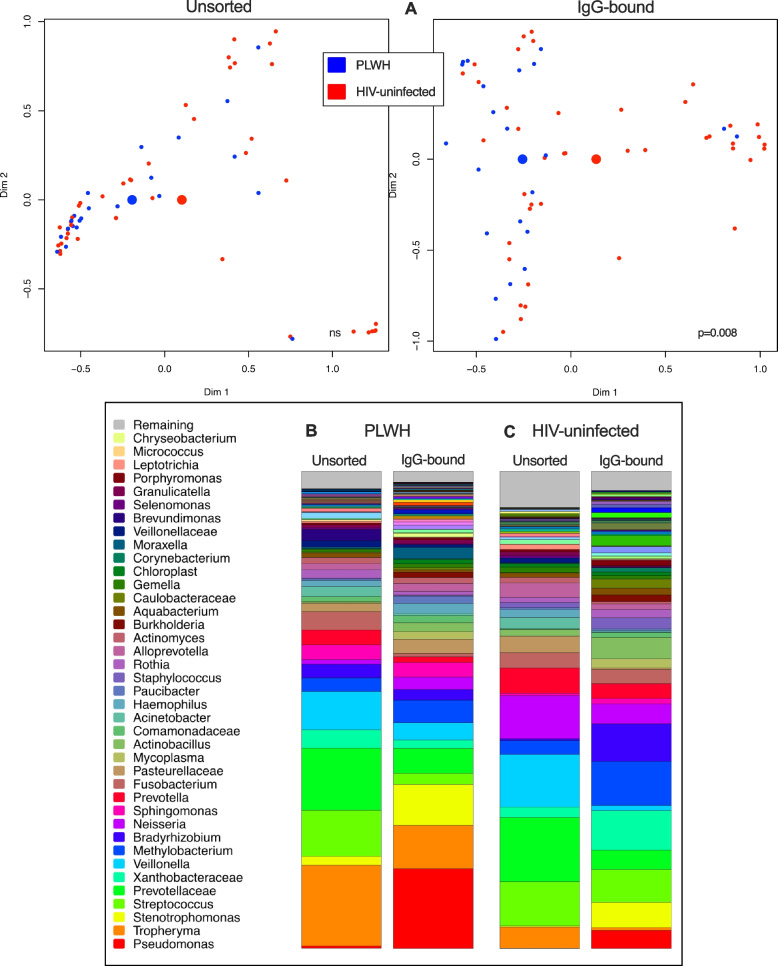
Bacterial taxa stratify by HIV status in IgG-bound BAL. **A** Unsorted (left) and IgG-bound (right) NMDS plots are displayed side by side to compare the differences in beta diversity observed when individuals are grouped by HIV status. We used Manhattan distance to visualize the sample clustering and performed a multivariate analysis of variance (PERMANOVA) to compare the bacterial communities in PLWH and HIV-uninfected individuals in both unsorted (raw) and IgG-bound BAL samples. Smaller dots signify the microbial community of individual samples, while the larger centroids represent the statistical average in each grouping (red = PLWH, blue = HIV uninfected). There were no taxonomic differences between PLWH and HIV-uninfected individuals in unsorted samples (*p* = 0.07); however, bacterial communities were stratified by HIV status in IgG-bound BAL samples (*p* = 0.008). **B** Stacked bar plots depict the relative abundance of bacteria in unsorted and IgG-bound BAL samples in PLWH. *Pseudomonas* was seen in greater abundance in IgG-bound samples when compared to unsorted samples. **C** Stacked bar plots depict the relative abundance of bacteria in unsorted and IgG-bound BAL samples in HIV-uninfected individuals

Unsorted and IgG-bound BAL microbial analysis from forty-two people living with HIV (PLWH) was then included to apply the technique to a representative disease state. Flow cytometry and qPCR demonstrated a higher quantity of IgG-bound bacteria in PLWH as compared to HIV-uninfected individuals (Additional file 1: Fig. S2). There was no difference in IgG concentration in the lungs of PLWH compared to HIV-uninfected individuals, but PLWH had high serum IgG level (*p* = 0.03, Additional file 1: Fig. S3). Next, we compared BAL microbial composition in PLWH and HIV-uninfected individuals. We employed a multivariate analysis, controlling for potentially confounding variables like sex, age, and smoking status. Similar to previous studies [3], we found no significant differences in the lung microbiota when comparing PLWH to HIV-uninfected individuals (*p* = 0.07, Fig. 2A). However, in IgG-bound samples, bacterial communities stratified by HIV status (PERMANOVA for Bray-Curtis distance, *p* = 0.008, *R*^2^ = 0.03; Fig. 2A). In PLWH, *Tropheryma* and *Prevotella* were the most abundant bacteria in raw BAL samples. In contrast, *Pseudomonas* was by far the most abundant IgG-bound bacteria in PLWH. In PLWH, *Pseudomonas* had greater relative abundance in IgG-bound BAL samples when compared to unsorted samples (Fig. 2B, Additional file 1: Fig. S7).

### Relationship between lung cytokine levels and IgG-bound bacteria

**Fig. 3:**
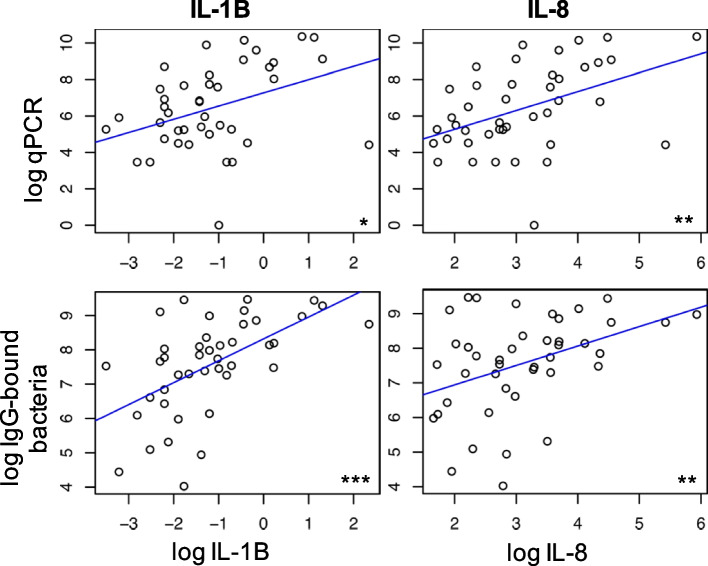
Increased IgG-bound bacteria in the lungs of PLWH correlate with BAL cytokine levels. We plotted IgG-bound bacteria as determined by qPCR (top) and flow cytometry (bottom) against BAL cytokine levels in PLWH. Individual dots represent the intercept between log-transformed cytokine level (*x*-axis) and log-transformed IgG-bound bacteria quantity (*y*-axis). A linear regression model was applied to assist with the visualization of the trend and quantify the degree of association. IL-8 and IL-1β correlated with increasing quantity of IgG-bound bacteria by qPCR (*p* = 0.003, *R*^2^ = 0.2 and *p* = 0.012, *R*^2^ = 0.15, respectively) and flow cytometry (*p* = 0.008, *R*^2^ = 0.16 and *p* < 0.001, *R*^2^ = 0.32, respectively)

To further explore an association between host recognition of the lung microbiome and inflammatory response, we quantified BAL cytokine levels and linked these to levels of IgG-bound bacteria. There was no correlation between cytokine levels with IgG-bound bacteria in HIV-uninfected individuals. We found that PLWH had significantly higher levels of BAL cytokines (Additional file 1: Fig. S3) as previously described [14]. In PLWH, higher levels of IgG-bound bacteria were associated elevated BAL cytokine levels including IL-8 and IL-1β by qPCR (*p* = 0.003, *R*^2^ = 0.2 and *p* = 0.012, *R*^2^ = 0.15, respectively) and flow cytometry (*p* = 0.008, *R*^2^ = 0.16 and *p* < 0.001, *R*^2^ = 0.32, respectively; Fig. 3).

## Discussion

Using a novel application of magnetic-activated cell sorting to isolate IgG-bound bacteria in the lung, we identified a distinct immunoglobulin-bound lung microbiota. Microbes bound by IgG were present in different relative abundance, resulting in markedly different community structure. Despite relatively high abundance of *Tropheryma* and oral anaerobes in unsorted samples, *Pseudomonas* was the most abundant bacteria recognized and bound by IgG. We saw differences in IgG-bound communities in a representative disease state in which few differences in the lung microbiome have been found with 16S sequencing. In contrast to IgA, which is the dominant immunoglobulin present in the mouth, IgG is more common in the lungs, which may be able to discriminate oral contaminants which make studying the lung microbiome more challenging. Application of the MACS technique may expand the investigation of the lung microbiome in health and disease.

Initial work with MACS has been applied to study bacteria in the gut and gastrointestinal disorders. For example, using a murine model, researchers demonstrated that the most “immunogenic” bacteria in the gut were bound by IgA, resulting in increased susceptibility to colitis in germ-free mice [4]. In a subsequent human study, IgG binding identified bacteria implicated in inflammatory bowel disease pathogenesis in pediatric patients [5].

In this first investigation applying MACS to the lung, we were able to isolate and quantify IgG-bound bacteria and reliably detect a different bacterial community structure than revealed by 16S rRNA gene sequencing of raw (unsorted) BAL. Although the typical oral bacteria identified in the lung microbiome were seen in both IgG-bound and raw BAL, there was relatively greater IgG-binding of *Pseudomonas*. Functional significance of immunoglobulin-bound bacteria is not known but suggests differences in the host response to similar bacteria and may contribute to previously defined “pneumotypes” [15], though we did not identify the cytokine signatures described by Segal et al.

It is plausible that immunoglobulin-bound bacteria play a role in chronic lung disease. Though traditionally considered protective, immunoglobulin binding of bacteria can lead to inflammation through opsonization [16] and antibody-dependent cell-mediated cytotoxicity [17]. B cells have been identified in greater abundance from the lungs of individuals with severe COPD [18] and have been implicated in emphysema pathogenesis [19, 20]. Host recognition of the lung microbiome corresponds to an inflammatory response [15]. Our results suggest that one possible mechanism of host recognition and response to the microbiome is through the humoral immune system.

We investigated HIV as a representative condition, a disease process that often leads to chronic lung disease even in the absence of significant tobacco abuse [21–23]. Prior investigation of the lung microbiome in HIV infection has failed to find significant taxonomic differences in individuals with normal CD4 counts on appropriate antiretroviral therapy [1, 3]; a somewhat unexpected finding given the differences in lung immune responses and pulmonary diseases in this population [21–25]. We were able to detect clear differences that were not seen in analyses of raw BAL. PLWH had greater abundance of IgG-bound respiratory pathogens such as *Pseudomonas* and *Stenotrophomonas*. We also found that elevated levels of multiple cytokines, including IL-6 and IL-1β, were correlated with higher numbers of IgG-bound bacteria. This correlation suggests that host recognition of lung microbiota may stimulate chronic lung inflammation, though future studies will be needed to further examine this association.

Interestingly, *Tropheryma* was identified in raw BAL samples in both PLWH and healthy (HIV-uninfected) controls, but was not nearly as abundant in IgG-bound communities. This bacteria has been consistently detected in the lung in both healthy populations and in PLWH [26]. Despite its presence in the lung, it has not been associated with pulmonary inflammation or lung function [27]. The discordance between its high abundance in raw samples and relatively lower abundance in IgG-bound samples could suggest that the absence of an inflammatory response to *Tropheryma* in the lungs may be due to a lack of active host recognition and response [26, 27].

Our study has several limitations. Given the low biomass within the lungs, any degree of experimental contamination can significantly skew results. We attempted to combat the impact of contamination by filtering all media, running UV light in the biosafety cabinet at the beginning and conclusion of each assay, and performing controls at each step of our experiment. We ran MACS controls, where 1% buffer was stained and run through the columns and subsequently sequenced. Using the sequencing data from the columns, we were able to subtract the 16S signal attributable to column contamination, reducing the amount of background noise. Additionally, controls were performed during each step of the PCR process. Another limitation was our use of only one antibody (IgG), without including analyses with IgA- and IgM-bound bacteria. We chose IgG as our initial analysis based on preliminary fluorescence-activated cell sorting data demonstrating the strongest signal (data not shown). Other variables such as smoking or prior lung infections could also impact the results. Notably, pack-year smoking history was one of several potentially confounding demographic variables that was included in PERMANOVA analyses in an attempt to control for these differences.

In conclusion, we report the first study of the immunoglobulin-bound lung microbiome. Using this technique, we identified distinct bacterial communities in the healthy lung and in PLWH and identified associations between IgG-bound bacteria and an inflammatory response. Though we chose to study immunoglobulin binding in HIV, adaptive immunity has been implicated in myriad chronic pulmonary diseases [18, 28, 29]. Host recognition and response to the lung microbiome can be applied to the investigation of other lung diseases, and modulation of immunoglobulin-binding of the lung and oral microbiome could be a potential therapeutic target in the future.

## Supplementary Information

**Additional file 1: Figure S1.** Flow cytometry confirming successful sorting with MACS. A representative BAL sample from one study participant, demonstrating adequate MACS sorting with >10-fold increase in “double-positive” FITC and PE, staining for bacterial DNA and IgG, respectively. The IgG-bound BAL fraction is depicted in top row, while IgG-unbound fraction is depicted in bottom row. **Figure S2.** Preliminary results from IgG-unbound fraction. Given the low biomass in our IgG-unbound samples, only the first 38 samples were sequenced and analyzed. There was no difference between PLWH (*n*=27) and HIV-uninfected individuals (*n*=11). *Pseudomonas*, *Stenotrophomonas*, *Bradyrhizium*, and *Streptococcus* were most abundant in both groups. **Figure S3.** Flow cytometry and qPCR data. Study participants were grouped by HIV status and then by use of anti-retroviral therapy (ART). **A**) Individuals were grouped by HIV status and groups compared using non-parametric t-testing (Mann U Whitney). PLWH had significantly more IgG-bound bacteria than HIV-uninfected individuals (*p*=0.0008). **B**) PLWH were then sub-divided by use of ART and compared with HIV-uninfected individuals. The three groups were compared using non-parametric t-tests (Mann U Whitney). PLWH not receiving ART had the highest abundance of IgG-bound bacteria by flow cytometry, when compared to HIV-uninfected individuals (*p*<0.0001) and PLWH taking ART (*p*=0.06). PLWH on ART also had greater abundance of IgG-bound bacteria (*p*=0.017). **C**) Quantitative PCR was used to quantify rRNA copy number in IgG-bound BAL samples. PLWH tended to have higher rRNA copy number (*p*=0.06). **Figure S4.** IgG quantification in blood and BAL. We measured BAL and serum IgG levels and compared levels between PLWH and HIV uninfected individuals. There was no significant difference in BAL concentration between individuals with and without HIV infections (**A**, *p*=0.07). PLWH had higher serum IgG concentration (**B**, *p*=0.03). **Fig. S5.** BAL cytokine levels. We measured BAL cytokines and compared concentration between PLWH and HIV uninfected individuals. Non-parametric t-testing (Mann U Whitney) was used to compare groups. Dots represent BAL cytokine concentration (pg/ml) in individual samples. PLWH had higher levels of several cytokines implicated in COPD pathogenesis, including TNF-α (*p*=0.03), IL-8 (*p*=0.03), IFN-γ (*p*=0.001), and MCP-1 (*p*=0.026). IL-6 and IL-1β levels tended to be higher in PLWH, though this was not statistically significant (*p*=0.07 and 0.052, respectively). **Figure S6.** Ranked ALR simple regression plots (HIV-uninfected). We applied a simple regression model (1=IgG-bound, 2=unsorted) to ranked bacteria by relative abundance using the additive log-ratio (ALR). *Prevotella*, *Veillonella*, and *Streptococcus* (*p*<0.001) were significantly more abundant in unsorted samples as compared to IgG-bound in HIV-uninfected individuals. **Figure S7.** Ranked ALR simple regression plots (PLWH). We applied a simple regression model (1=IgG-bound, 2=unsorted) to ranked bacteria by relative abundance using the additive log-ratio (ALR). *Prevotella*, *Veillonella*, and *Streptococcus* (*p*<0.001) were significantly more abundant in unsorted samples as compared to IgG-bound in PLWH. *Pseudomonas* was significantly more abundant in IgG-bound samples (*p*<0.001).

## Data Availability

Data sets generated and analyzed during the current study are available in the NIH Sequence Read Archive (SRA). Sequencing data was uploaded to the NIH Sequence Read Archive (SRA), PRJNA720126 [https://www.ncbi.nlm.nih.gov/sra/?term=PRJNA720126].
