# Supplementary material for: Magnetic-activated cell sorting identifies a unique lung microbiome community
Source: Microbiome. 2023 May 25;11:117. doi: 10.1186/s40168-022-01434-5 (PMC10210470; doi:10.1186/s40168-022-01434-5)

**Supplement 1 – Flow cytometry confirming successful sorting with MACS**

A representative BAL sample from one study participant, demonstrating adequate MACS sorting with >10-fold increase in “double-positive” FITC and PE, staining for bacterial DNA and IgG, respectively. The IgG-bound BAL fraction is depicted in top row, while IgG-unbound fraction is depicted in bottom row.

**Supplement 2 – Preliminary results from IgG-unbound fraction**

Given the low biomass in our IgG-unbound samples, only the first 38 samples were sequenced and analyzed. There was no difference between PLWH (n=27) and HIV-uninfected individuals (n=11). *Pseudomonas*, *Stenotrophomonas*, *Bradyrhizium*, and *Streptococcus* were most abundant in both groups.

**
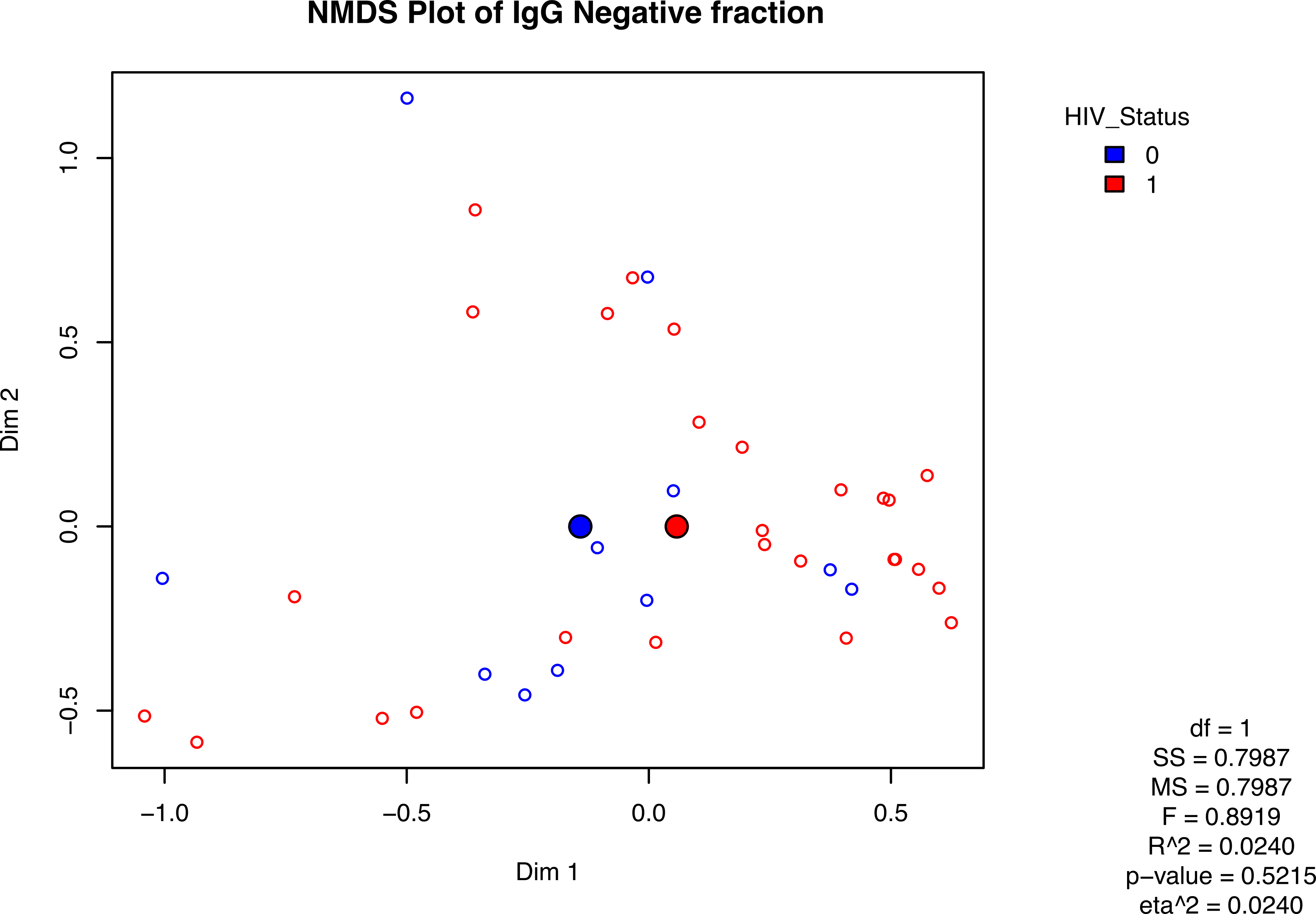
**

**
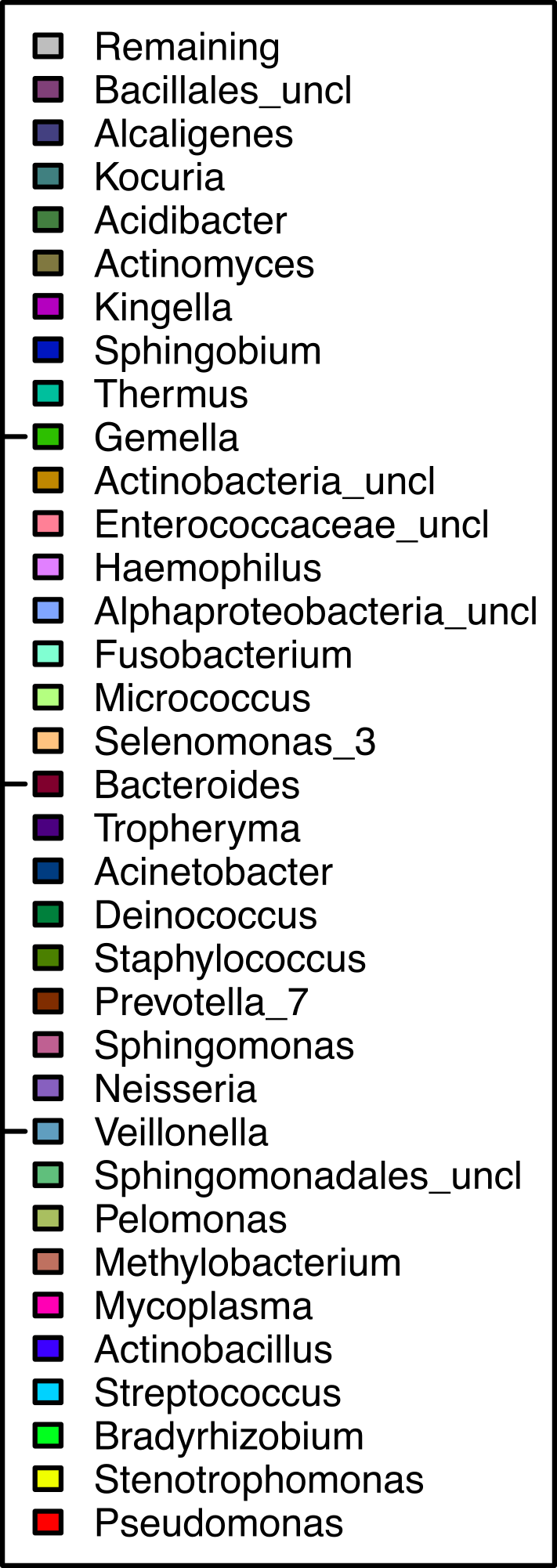

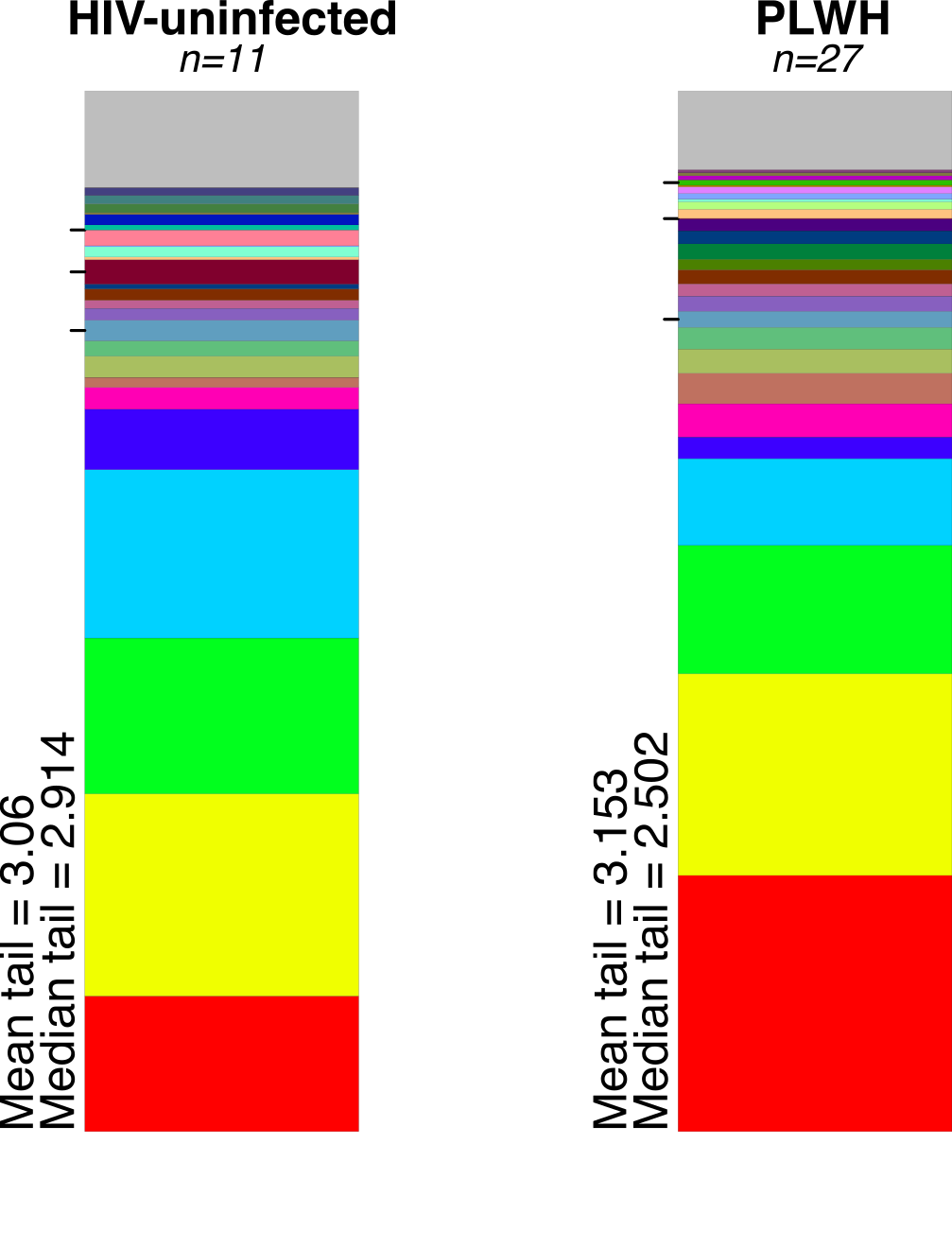
**

**Supplement 3 – Flow cytometry and qPCR data**

Study participants were grouped by HIV status and then by use of anti-retroviral therapy (ART). **A)** Individuals were grouped by HIV status and groups compared using non-parametric t-testing (Mann U Whitney). PLWH had significantly more IgG-bound bacteria than HIV-uninfected individuals (p=0.0008). **B)** PLWH were then sub-divided by use of ART and compared with HIV-uninfected individuals. The three groups were compared using non-parametric t-tests (Mann U Whitney). PLWH not receiving ART had the highest abundance of IgG-bound bacteria by flow cytometry, when compared to HIV-uninfected individuals (p<0.0001) and PLWH taking ART (p=0.06). PLWH on ART also had greater abundance of IgG-bound bacteria (p=0.017). **C)** Quantitative PCR was used to quantify rRNA copy number in IgG-bound BAL samples. PLWH tended to have higher rRNA copy number (p=0.06).

**Supplement 4 – IgG quantification in blood and BAL**

We measured BAL and serum IgG levels and compared levels between PLWH and HIV uninfected individuals. There was no significant difference in BAL concentration between individuals with and without HIV infections (A, p=0.07). PLWH had higher serum IgG concentration (B, p=0.03).

A.
B.

**Supplement 5 – BAL cytokine levels**

We measured BAL cytokines and compared concentration between PLWH and HIV uninfected individuals. Non-parametric t-testing (Mann U Whitney) was used to compare groups. Dots represent BAL cytokine concentration (pg/ml) in individual samples. PLWH had higher levels of several cytokines implicated in COPD pathogenesis, including TNF-α (p=0.03), IL-8 (p=0.03), IFN-γ (p=0.001), and MCP-1 (p=0.026). IL-6 and IL-1β levels tended to be higher in PLWH, though this was not statistically significant (p=0.07 and 0.052, respectively).

**Supplement 6 – Ranked ALR simple regression plots (HIV-uninfected)**

We applied a simple regression model (1=IgG-bound, 2=unsorted) to ranked bacteria by relative abundance using the additive log-ratio (ALR). *Prevotella*, *Veillonella*, and *Streptococcus* (p<0.001) were significantly more abundant in unsorted samples as compared to IgG-bound in HIV-uninfected individuals.


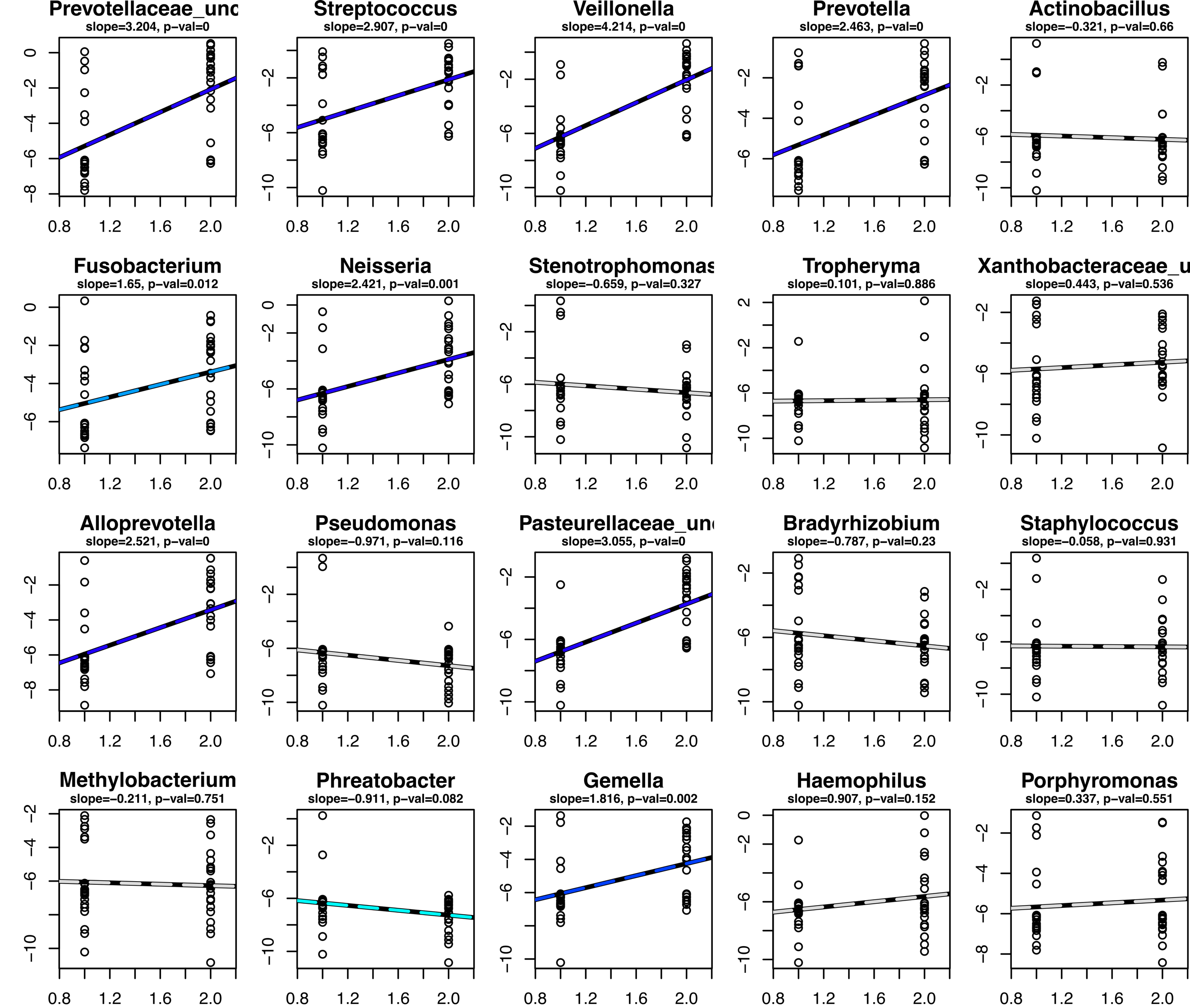


**Supplement 7 – Ranked ALR simple regression plots (PLWH)**

We applied a simple regression model (1=IgG-bound, 2=unsorted) to ranked bacteria by relative abundance using the additive log-ratio (ALR). *Prevotella*, *Veillonella*, and *Streptococcus* (p<0.001) were significantly more abundant in unsorted samples as compared to IgG-bound in PLWH. *Pseudomonas* was significantly more abundant in IgG-bound samples (p<0.001).


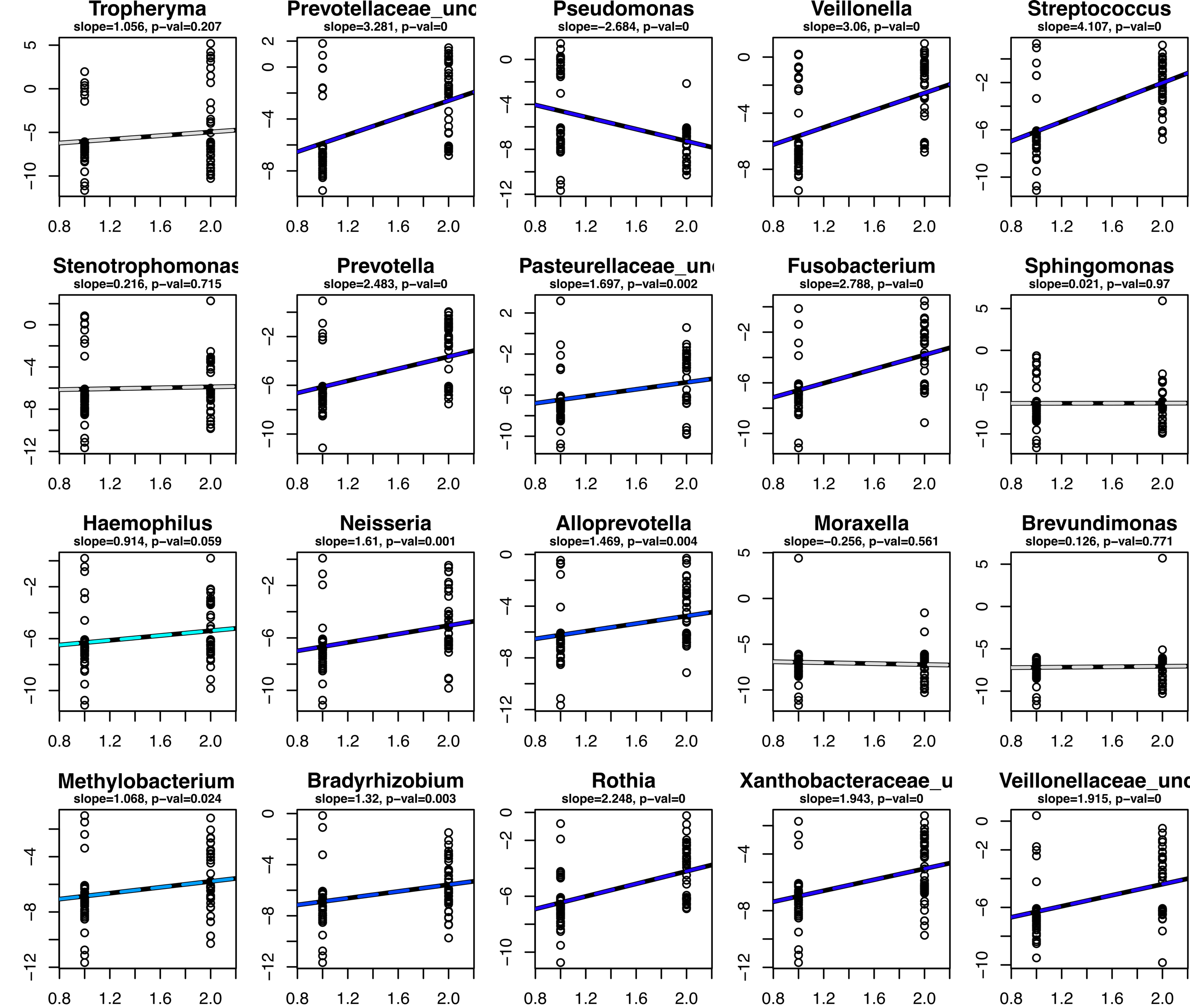

Supplement: Supplementary file 2 — Additional file 1: Figure S1. Flow cytometry confirming successful sorting with MACS. A representative BAL sample from one study participant, demonstrating adequate MACS sorting with >10-fold increase in “double-positive” FITC and PE, staining for bacterial DNA and IgG, respectively. The IgG-bound BAL fraction is depicted in top row, while IgG-unbound fraction is depicted in bottom row. Figure S2. Preliminary results from IgG-unbound fraction. Given the low biomass in our IgG-unbound samples, only the first 38 samples were sequenced and analyzed. There was no difference between PLWH (n=27) and HIV-uninfected individuals (n=11). Pseudomonas, Stenotrophomonas, Bradyrhizium, and Streptococcus were most abundant in both groups. Figure S3. Flow cytometry and qPCR data. Study participants were grouped by HIV status and then by use of anti-retroviral therapy (ART). A) Individuals were grouped by HIV status and groups compared using non-parametric t-testing (Mann U Whitney). PLWH had significantly more IgG-bound bacteria than HIV-uninfected individuals (p=0.0008). B) PLWH were then sub-divided by use of ART and compared with HIV-uninfected individuals. The three groups were compared using non-parametric t-tests (Mann U Whitney). PLWH not receiving ART had the highest abundance of IgG-bound bacteria by flow cytometry, when compared to HIV-uninfected individuals (p<0.0001) and PLWH taking ART (p=0.06). PLWH on ART also had greater abundance of IgG-bound bacteria (p=0.017). C) Quantitative PCR was used to quantify rRNA copy number in IgG-bound BAL samples. PLWH tended to have higher rRNA copy number (p=0.06). Figure S4. IgG quantification in blood and BAL. We measured BAL and serum IgG levels and compared levels between PLWH and HIV uninfected individuals. There was no significant difference in BAL concentration between individuals with and without HIV infections (A, p=0.07). PLWH had higher serum IgG concentration (B, p=0.03). Fig. S5. BAL cytokine levels. We measured BAL cyt [file 40168_2022_1434_MOESM1_ESM.docx]
